# Harnessing the Activation of RIG-I Like Receptors to Inhibit Glioblastoma Tumorigenesis

**DOI:** 10.3389/fnmol.2021.710171

**Published:** 2021-07-08

**Authors:** Francesca Bufalieri, Irene Basili, Lucia Di Marcotullio, Paola Infante

**Affiliations:** ^1^Department of Molecular Medicine, University La Sapienza, Rome, Italy; ^2^Laboratory affiliated to Istituto Pasteur Italia-Fondazione Cenci Bolognetti, Rome, Italy; ^3^Center For Life Nano Science@Sapienza, Istituto Italiano di Tecnologia, Rome, Italy

**Keywords:** glioblastoma, immunotherapy, RIG-I like receptors (RLRs), RIG-I, MDA5, LGP2, RLRs agonists

## Abstract

Glioblastoma (GB) is an incurable form of brain malignancy in an adult with a median survival of less than 15 months. The current standard of care, which consists of surgical resection, radiotherapy, and chemotherapy with temozolomide, has been unsuccessful due to an extensive inter- and intra-tumoral genetic and molecular heterogeneity. This aspect represents a serious obstacle for developing alternative therapeutic options for GB. In the last years, immunotherapy has emerged as an effective treatment for a wide range of cancers and several trials have evaluated its effects in GB patients. Unfortunately, clinical outcomes were disappointing particularly because of the presence of tumor immunosuppressive microenvironment. Recently, anti-cancer approaches aimed to improve the expression and the activity of RIG-I-like receptors (RLRs) have emerged. These innovative therapeutic strategies attempt to stimulate both innate and adaptive immune responses against tumor antigens and to promote the apoptosis of cancer cells. Indeed, RLRs are important mediators of the innate immune system by triggering the type I interferon (IFN) response upon recognition of immunostimulatory RNAs. In this mini-review, we discuss the functions of RLRs family members in the control of immune response and we focus on the potential clinical application of RLRs agonists as a promising strategy for GB therapy.

## Introduction

Glioblastoma (GB) is the most frequent and aggressive primary adult brain tumor, with a median overall survival (OS) of approximately 1 year (Ostrom et al., 2014). First-line therapy for newly diagnosed GB consists of maximal surgical resection of the tumor, followed by radiotherapy (RT) and concomitant adjuvant chemotherapy with the alkylating agent Temozolomide (TMZ; Stupp et al., 2005, 2009). Unfortunately, the disease remains incurable and often returns as recurrent GB (Lieberman, 2017). Limited progress in the development of more effective therapeutic approaches for GB is mostly due to its heterogeneous genetic, molecular landscape, and cell plasticity (Brennan et al., 2013; Meyer et al., 2015; Gangoso et al., 2021).

GB can be subdivided into primary and secondary subtypes on the basis of their clinical presentation (Dunn et al., 2012). Despite these GB subtypes being morphologically and clinically indistinguishable and sharing the same devastating prognosis, they revealed differences in genetic alterations, such as gene copy number aberrations, changes in chromosome structure, and genetic instability (Cancer Genome Atlas Research Network, 2008). Multiple genetic drivers are involved in the onset of GB, including amplification of epidermal growth factor receptor (*EGFR*) gene and mutations in telomerase reverse transcriptase (*TERT*), isocitrate dehydrogenase (*IDH*), phosphatase and tensin homolog (*PTEN*), platelet-derived growth factor receptor alpha (*PDGFRα*), and neurofibromatosis type 1 (*NF1*) genes (Stoyanov and Dzhenkov, 2018). Based on this evidence, several targeted therapies for GB have been tested, such as against growth factor receptors (i.e., EGFR) and downstream pathways (i.e., PI3K/AKT/mTOR and MAPK; Le Rhun et al., 2019). However, these approaches have shown only occasional responses in patients, and none of them has been formally validated as effective in clinical trials (Touat et al., 2017). Furthermore, current clinical immunotherapy strategies have largely been disappointing (Weenink et al., 2020; Medikonda et al., 2021), due to the presence of tumor immunosuppressive microenvironment. For this reason, the activation of an anticancer innate immunity in the tumor microenvironment (TME) stands as an emerging option to increase immunogenicity also in GB (Elion and Cook, 2018).

Host innate immunity represents the first line of defense and mediates the detection of danger signals through pattern recognition receptors (PRRs), thereby modulating the expression of cytokines and chemokines that recruit T-lymphocytes to the affected tissue, increasing antigen presentation and cross-priming to antigen-specific T-cells (Takeuchi and Akira, 2010; Shalapour and Karin, 2015).

Retinoic acid-inducible gene I (RIG-I)-like receptors (RLRs) belong to the PRRs family and they are key sensors of both viral and host-derived RNAs, thus mediating the activation of the innate immune system through the type I interferon (IFN) response (Rehwinkel and Gack, 2020).

Numerous findings have underlined that the activation of RLRs signaling in the tumor triggers several effects: (i) tumor cell death; (ii) activation of innate immune cells in the TME; (iii) increased recruitment and cross-priming of adaptive immune effectors, especially in poorly immunogenic, non-T-cell inflamed tumors. These evidences have brought out the possibility to target RLRs for anti-cancer therapy. To date, synthetic RLRs mimetics are under investigation in pre-clinical and early clinical studies for the treatment of gliomas, multiple myeloma, breast, pancreatic, and ovarian cancers (Sabbatini et al., 2012; Okada et al., 2015; Dillon et al., 2017; Mehrotra et al., 2017).

In this mini-review we provide an overview of immunotherapeutic approaches tested in GB patients and discuss the role of RLR family members in the control of the immune response, focusing on the recent developments of RLRs mimetics as new therapeutic perspectives for GB therapy.

## Brief Overview of Immunotherapeutic Approaches in Glioblastoma

Clinical use of immunotherapies for brain tumors represents a great challenge particularly due to the blood–brain barrier and the unique immune tumor microenvironment afforded by the central nervous system (CNS)-specific cells. Recently, the discovery of the CNS lymphatic system eroded the concept of CNS as an “immune privileged site”, thus prompting the translation of immunotherapy to brain malignancies (Aspelund et al., 2015; Louveau et al., 2015). In GB, four main immunotherapeutic approaches are under investigation in clinical trials: (i) immune checkpoint inhibitors (ICIs); (ii) vaccination with peptides or dendritic cells (DCs); (iii) adoptive transfer of effector lymphocytes; and (iv) oncolytic virotherapy (Weenink et al., 2020; Majc et al., 2021).

ICIs strategy is based on the use of monoclonal antibodies able to block receptors (i.e., PD-1) or their ligands (i.e., PD-L1) expressed by immune cells and tumor cells to elicit an effective antitumor immune CD8^+^ T cell response (Darvin et al., 2018). ICIs have been extensively studied for GB, used either as monotherapy or in combination with RT and TMZ. CheckMate 143 (NCT02017717) was the first phase III trial to evaluate the efficacy of anti-PD1 nivolumab vs. Vascular Endothelial Growth Factor (VEGF)-inhibitor bevacizumab in patients with recurrent GB. Overall survival (OS) of patients was not improved after treatment with nivolumab compared to bevacizumab, with a median survival time of 9.8 and 10.0 months, respectively (Reardon et al., 2020a).

Currently, two ongoing phase III clinical trials, Checkmate 498 (NCT02617589) and Checkmate 548 (NCT02667587), are evaluating the effectiveness of anti-PD-1 and RT combined therapy, with and without TMZ, in patients with newly diagnosed O-6-methylguanine DNA methyltransferase (MGMT)-unmethylated GB or MGMT-methylated GB, respectively (Lim et al., 2017). Safety analysis from these trials showed that these combinations are well-tolerated, but OS does not appear to increase in treated patients.

Interestingly, two recent Phase II clinical trials based respectively on the administration of the anti-PD-1 antibody nivolumab before and after surgery (NCT02550249), and on the use of the anti-PD-L1 antibody durvalumab in combination with RT (NCT02336165), have shown promising results prolonging the OS of GB patients (Reardon et al., 2019; Schalper et al., 2019). These results prompted to focus attention on the development of combinatorial therapy of ICIs with RT and/or chemotherapy as well as on alternative immunotherapeutic strategies for GB. Therapeutic cancer vaccines aim to induce an anti-tumor immune response through the exogenous administration of selected tumor-associated antigens, combined with adjuvants that activate DCs, or even DCs themselves (Saxena et al., 2021).

The variant III of EGFR (EGFRvIII) is a constitutively active mutant of EGFR expressed on GB cells in 25–30% of patients and it represents a recognized target in many peptide vaccination studies (Congdon et al., 2014). A peptide vaccine targeting EGFRvIII (rindopepimut) was evaluated in GB patients following gross total resection and chemo-radiotherapy, showing a modest improvement of OS (David et al., 2015). However, the multicenter phase III trial ACT IV failed to confirm this initial result and was terminated after interim analysis (Weller et al., 2017).

A phase II trial known as ReACT found that the treatment with rindopepimut plus bevacizumab of patients with recurrent GB increased the OS to 12.0 months compared to 8.8 months with bevacizumab plus vaccine control treatment (Reardon et al., 2020b). While rindopepimut has elicited immune responses and may have some activity in a small and selected cohort of recurrent GB patients, further studies are required to determine the optimal patient population and treatment regimen.

Dendritic cells (DCs) vaccines are based on the use of engineered DCs loaded with tumor-specific antigen(s) with the aim of activating antigen-specific T-cells that selectively eliminate antigen-bearing tumor cells (Van Willigen et al., 2018). A randomized phase-III trial based on the use of autologous tumor lysate-pulsed DC vaccine (DCVax-L) in addition to Stupp protocol in patients with newly diagnosed glioblastoma led to the approval of this vaccine in Switzerland (Liau et al., 2018).

The high heterogeneity of GB has highlighted the need to identify personalized treatments. In this regard, particular interest was given to chimeric antigen receptor (CAR) T cell therapy, based on the use of genetically modifying T cells harvested from the patients. A phase I trial (NCT01109095) of Human Epidermal Growth Factor Receptor 2 (HER-2)-targeted CAR-T cell therapy has shown an acceptable safety profile and some patients have demonstrated stable disease for 8 weeks–29 months (Ahmed et al., 2017). However, one of the biggest pitfalls of this approach remains the difficulty to develop a CAR-T cell therapy that can target all of the clonal populations of GB (Fecci and Sampson, 2019). Pre-clinical results suggest that the use of tri-valent CAR-T cells able to target multiple tumor-specific antigens (i.e., HER2, IL13Rα2, and EphA2) may be more efficacious than mono- or bi-valent CAR-T cells therapy (Bielamowicz et al., 2017).

Further clinical studies are also needed to confirm the efficacy of oncolytic virus therapy in GB. This strategy is based on the intratumoral administration of genetically modified viruses that usually have the ability to selectively replicate inside malignant infected cells. The replication of lytic viruses leads to the destruction of the target cell and further propagation of viral progeny, which can induce an anti-tumor immune response. Some tumor-selective lytic viruses, such as herpes simplex virus, adenovirus, and poliovirus have shown the ability to replicate in GB cells, but they are currently being studied in early phase clinical trials (Martikainen and Essand, 2019).

An urgent aspect that needs to be considered in the further progression of immunotherapy for GB is the tumor microenvironment (TME), which has an influence on tumor initiation, response, and therapy. Immunotherapeutic strategies, including ICIs or CAR-T cells, are specifically aimed at enhancing adaptive anti-tumor immunity. However, these approaches appear less effective in cancers that are poorly immunogenic, showing low levels of tumor-infiltrating lymphocytes (TILs), minimal cross-presentation of tumor neo-antigens, or high levels of immune-suppressive leukocytes tumor-associated macrophages (TAMs; Marincola et al., 2000; Garrido and Algarra, 2001; Woroniecka et al., 2018).

All these findings recommend future treatment strategies that sensitize GB to immunotherapies, through the activation of either adaptive or innate immune response, or combinatorial immunotherapeutic approaches able to hit this tumor at multiple levels.

## RLRs: Members and Mechanisms of Activation

The RIG-I like receptors (RLRs) are a protein family of cytoplasmic viral RNA detectors composed of three members: RIG-I (Retinoic acid Inducible Gene 1), MDA5 (melanoma differentiation associated factor 5), and LGP2 (laboratory of genetics and physiology 2; Loo and Gale, 2011; Onoguchi et al., 2011).

**Figure 1:**
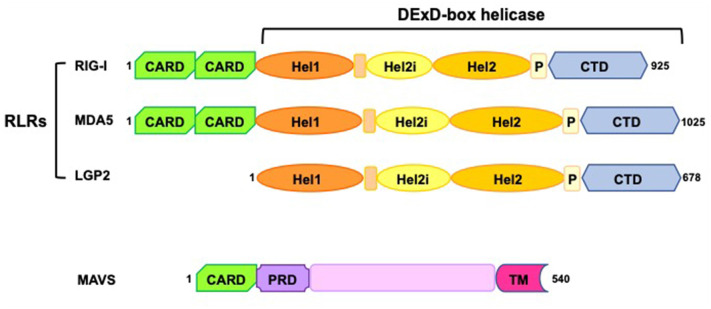
Schematic representation of RIG-I-like receptors (RLRs) and mitochondrial antiviral-signaling protein (MAVS) domains. RLRs have a central DExD-box helicase containing: (i) two conserved helicase domains (Hel1 and Hel2); (ii) a helicase insertion domain (Hel2i) with ATPase activity; (iii) a pincer domain (P); (iv) a C-terminal domain (CTD). Both the helicase domains and the CTD have RNA binding ability. RIG-I and MDA5 have two N-terminal caspase activation and recruitment domains (CARDs), essential for the interaction with MAVS and the induction of downstream signaling. MAVS consists of a single CARD, a proline-rich region (PRD), and a C-terminus transmembrane domain (TM) required for its tethering to mitochondria, mitochondrial-associated membranes (MAM), and peroxisomes.

All RLRs are characterized by a conserved structure, consisting of a central DExD/H box RNA helicase domain with ATPase activity and a carboxy-terminal domain (CTD), which plays a crucial role in detecting immunostimulatory RNAs (Figure 1). In addition, the CTD of RIG-I and LGP2 acts as a repressor domain (RD), keeping the two receptors in an inactive form in the absence of stimuli (Loo and Gale, 2011; Onoguchi et al., 2011; Agier et al., 2018). Further, both RIG-I and MDA5 have additional amino-terminal caspase activation and recruitment domains (CARDs) that mediate downstream signaling (Figure 1). LGP2 lacks the CARDs and it is widely considered a regulator of RLRs signaling rather than an active receptor, exerting co-stimulatory and inhibitory functions on MDA5 and RIG-I, respectively (Gack, 2014; Reikine et al., 2014; Rehwinkel and Gack, 2020).

RLRs are expressed in a wide range of tissues and they commonly mediate the activation of the innate immune system by triggering type I interferon (IFN) response and induce apoptosis upon recognition of RNAs not usually present in healthy cells (Loo and Gale, 2011; Reikine et al., 2014; Rehwinkel and Gack, 2020).

RIG-I and LGP2 are physiologically found in an auto-repressed state and are activated by the presence of immunostimulatory RNAs that induce their conformational change leading to the binding with ATP (Kowalinski et al., 2011; Bruns et al., 2013). Compared to RIG-I and LGP2, MDA5 shows a more open structural conformation even in the absence of RNA ligands (Berke and Modis, 2012; Brisse and Ly, 2019).

Although RNAs activating RLRs are generally of viral origin, RNAs that are unusual, mislocalized, or misprocessed can activate these receptors (Rehwinkel and Gack, 2020).

RIG-I recognizes short 5′ tri-phosphorylated double-strand RNAs (dsRNAs), single-strand RNAs (ssRNAs) forming secondary structure (i.e., hairpin or panhandle conformations), and RNAs with uncapped diphosphate (PP) groups at the 5′ (Hornung et al., 2006; Pichlmair et al., 2006; Schmidt et al., 2009; Schlee, 2013; Goubau et al., 2014). In order to allow the recognition by RIG-I, the 5′-terminal nucleotide of activating RNAs must not be methylated at the 2′-O position. Indeed, this modification is a crucial hallmark of endogenous RNAs. The steric exclusion of N1–2′O-methylated RNA mediated by the conserved amino acid H830 in the RIG-I RNA binding pocket prevents RIG-I stimulation by self-RNAs (Schuberth-Wagner et al., 2015).

MDA5 and LGP2 bind to long dsRNAs, however, their activating RNAs are less characterized (Hornung et al., 2006; Pichlmair et al., 2009; Schlee, 2013).

Overall, the activation of RLRs is a multi-step process that can be triggered not only by viral RNAs, but also by different regulatory mechanisms that modulate the amount and the activity of these receptors, as well as other components of the RLRs signaling cascade, such as the mitochondrial antiviral-signaling protein (MAVS, also named IPS-1, VISA, or CARDIF; Gack, 2014; Rehwinkel and Gack, 2020).

## RLRs: Downstream Signaling and Functions

Upon ligand recognition, RIG-I and MDA5 interact through their CARDs with MAVS, an adaptor protein needed to initiate the RLRs signaling cascade. Given that LGP2 does not have a CARD, it does neither recruit MAVS nor induce MAVS signaling (Reikine et al., 2014).

MAVS protein belongs to the 14–3–3 protein family, containing a transmembrane domain by which it tethers the intracellular membrane of mitochondria, mitochondrial-associated membranes, and peroxisomes, thereby regulating the RLRs signaling transduction from the cytosol to mitochondria (Gack, 2014; Rehwinkel and Gack, 2020). Importantly, MAVS signaling is dependent on cellular localization, inducing a different antiviral response depending on whether its activation occurs at the peroxisomal or mitochondrial membrane (Dixit et al., 2010; Yu et al., 2010).

At an early time of viral infection, MAVS activates the cytosolic TANK-binding kinase 1 (TBK1) and IκB kinase-ε (IKKε). This event leads to the activation of the transcriptional factors IFN regulatory factor 1 (IRF1) and 3 (IRF3) triggering an immediate IFN-independent signaling that induces a rapid expression of several antiviral or immunostimulatory genes (i.e., IFN-stimulated genes, ISGs).

**Figure 2:**
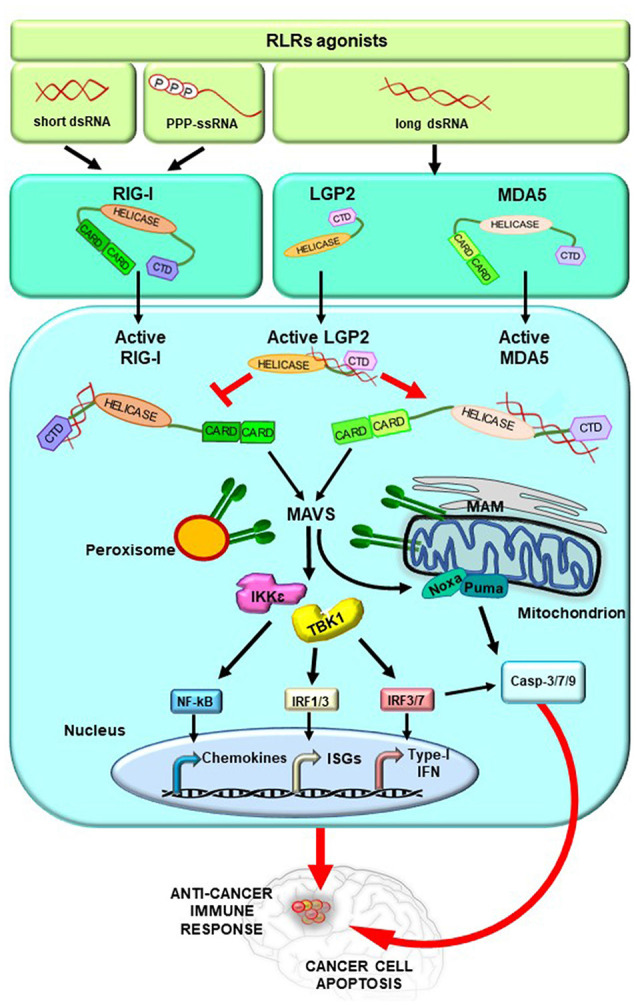
RLRs signaling pathway activation. Upon binding of immunostimulatory RNAs, RLRs undergo conformational changes leading them to an active state. These events are tightly regulated by ubiquitylation and phosphorylation processes (not shown for simplicity) and by the action of LGP2, which stimulates MDA5 and inhibits RIG-I. The activation of RIG-I and MDA5 induces the exposition of their CARDs for the interaction with MAVS. MAVS is located on mitochondria, mitochondrial-associated membranes (MAM), and peroxisomes and transduces the signal to TANK-binding kinase 1 (TANK1) and IkB Kinase ε (IKKε). Subsequently, the interferon regulatory factors 1, 3, and 7 (IRF1, 3, and 7) are activated together with the transcription factor nuclear factor-κB (NF-κB), leading to the expression of type I interferon (IFN) and other genes, such as IFN-stimulated genes (ISGs). In addition, activated MAVS promotes intrinsic/mitochondrial apoptosis through the expression of the pro-apoptotic genes *Noxa* and *Puma*, whose encoded proteins finally induce the cleavage of the caspases 3, 7, and 9 into the active forms. Overall, these molecular events stimulate anticancer immune response and lead to cancer cell apoptosis.

Conversely, at a later time from infection, mitochondrial MAVS promotes an IFN-dependent signaling pathway activating IRF3/7, which together with nuclear factor-κB (NF- κB) induce the transcription of type I IFNs and ISGs (Dixit et al., 2010; Gack, 2014; Rehwinkel and Gack, 2020; Figure 2).

Interestingly, studies from different research groups have reported that the activation of RLRs, triggered by the cytosolic delivery of RNA ligands, requires the function of MAVS and IRF3 leading either to type I IFN production or to intrinsic apoptosis pathway through the expression of the pro-apoptotic genes *Noxa* and *Puma* (Poeck et al., 2008; Rintahaka et al., 2008; Besch et al., 2009; Maelfait et al., 2020; Figure 2).

The complexity of the RLRs signaling is finely orchestrated by several combinatorial mechanisms that ensure an appropriate immune response in presence of immunostimulatory RNAs and preserve immune homeostasis under normal physiological conditions. Among these regulatory mechanisms, both non-degradative and degradative ubiquitylation events, deubiquitylation and phosphorylation processes are the most studied post-translational modifications of the RLRs (Reikine et al., 2014; Chan and Gack, 2015; Rehwinkel and Gack, 2020).

Over the past few years, RLRs activation has also been observed in several autoinflammatory and autoimmune diseases, as well as in cancer regardless of viral infection (Rehwinkel and Gack, 2020).

Despite this activation stimulating the immune response, cancer cells are able to escape the immunosurveillance by the selection of non-immunogenic tumor cell variants or by active immunosuppression (Zitvogel et al., 2008). In this regard, the RLRs stimulation could represent an alternative therapeutic approach to overcome tumor-mediated immunosuppression in relation to their role in stimulating type I IFN production and apoptosis (Zitvogel et al., 2015). Recently, promising data have been obtained with the use of specific RIG-I and MDA5 agonists as vaccine adjuvant or potentiator in cancer immunotherapies, giving the possibility to exploit the patient’s immune defenses (Kasumba and Grandvaux, 2019).

## Agonists of RLRs as a Promising Therapeutic Strategy for Glioblastoma

Over the past decade, the scientific community’s interest in the understanding of the multiple biological functions of RLRs has particularly been focused on the study of their non-infectious activation as a possible cancer treatment option. These innovative therapeutic approaches aim to improve the expression and the activity of RLRs in order to stimulate innate and adaptive immune responses against tumor cells. In this regard, the use of replication incompetent (oncolytic) viruses and synthetic RLR agonists as anticancer agents are being investigated in a wide range of tumors, including GB (Wu et al., 2017; Elion and Cook, 2018; Iurescia et al., 2020).

The effectiveness of the standard treatments in GB is limited due to the remarkable tumor heterogeneity and the difficulty of eradicating GSCs, which contribute to a large extent to the rapid proliferation of cancer cells, therapeutic resistance, and immune attenuation (DeCordova et al., 2020; Majc et al., 2021). For this reason, the use of immunotherapeutic approaches based on the activation of RLRs could be a good strategy for the treatment of GB, with the aim to increase tumor cell death *via* mitochondrial apoptosis and to overcome the obstacle of immunosuppression.

Glas et al. (2013) provided the first *in vitro* evidence on the potential benefit of the use of RLRs agonists to counteract tumor growth in GB, demonstrating that the induction of the immune response through the activation of RIG-I and MDA5 targets different populations of GB cells. Specifically, the stimulation of these receptors by polyinosinic-polycytidylic acid [p(I:C)] and 5′-triphosphate RNA (3pRNA) in human primary GB (pGB) leads to the activation of the innate immune system [evaluated by the secretion of the in C-X-C motif chemokine ligand-10 (CXCL10) and type I IFN], and to the induction of apoptosis. Interestingly, the authors found that the treatment with p(I:C) and 3pRNA target tumor cells with and without stem cell feature to the same extent, having only mild toxicity in human non-malignant neural cells (Glas et al., 2013).

The expression level of RIG-I or MDA5 in GB is another important aspect to be considered for the clinical application of this therapeutic option. Glas et al. (2013) reported that pGB CSCs have low baseline expression levels of these receptors, which can be increased through the treatment with IFN-β.

Accordingly, a recent study has shown very low protein levels of RIG-I in GB specimens as well as in different human GB cell lines when compared to healthy brain tissue and non-tumor brain cell lines, respectively (Bufalieri et al., 2020). In this study, the authors have demonstrated that RIG-I protein levels are inversely associated with the expression of the RNA-binding Ubiquitin Ligase MEX3A, known to play an oncogenic role in several tumors (Jiang et al., 2012; Huang et al., 2017; Liang et al., 2020; Wang et al., 2020, 2021; Wei et al., 2020).

Remarkably, it has been found that MEX3A binds and ubiquitylates RIG-I, thus promoting its proteasomal degradation. Of note, the genetic depletion of MEX3A leads to an increase of RIG-I protein levels in GB and to a reduction of tumor growth, although it is still under investigation whether this effect is mediated by a consequent activation of RIG-I (Bufalieri et al., 2020).

These findings suggest that targeting these receptors with specific agonists could lead to strong activation of the immune response and induction of apoptosis in tumor cells. Particularly, the anticancer effects promoted by the stimulation of RIG-I/MDA5/MAVS signaling are triggered by the release of type I IFNs, chemokines, and pro-inflammatory cytokines. This event results in cancer cell apoptosis either by IFN-dependent or IFN-independent manner. In addition, the production of chemokines and cytokines by RIG-I/MDA5 in TME activates several innate immune effectors, such as NK cells and macrophages, and increases the recruitment and the cross-priming of adaptive immune effectors (e.g., CD8^+^ T-lymphocytes), while reducing the T-regulatory cell differentiation. The maturation and activation of antigens-presenting cells (e.g., macrophages and DCs) result in an increased presentation of cancer-associated antigens to CD8^+^ T cells, which leads to cancer antigen-specific cytotoxicity (Wu et al., 2017; Elion and Cook, 2018; Iurescia et al., 2020). In the light of these evidence, RLRs mimetics for the treatment of GB could be also used to enhance the efficacy of other immunomodulatory drugs. In this regard, new therapeutic approaches that combine the use of RLRs agonists and ICIs have already shown good results in pre-clinical and clinical studies (i.e., NCT03065023; NCT03739138; NCT03203005; NCT03291002) for the treatment of different tumors (Elion et al., 2018; Middleton et al., 2018; Elion and Cook, 2019; Meister et al., 2019).

Several studies have reported that radiotherapy activates type I IFN production, indicating that the IFN signaling plays an important role in the tumor cytotoxicity and/or the activation of the immune response induced by the ionizing radiation (IR) treatment (Tsai et al., 2007; Chajon et al., 2017; Van Limbergen et al., 2017; Turgeon et al., 2019). In particular, RIG-I is essential for the cytotoxic IFN-β response and apoptosis induced by IR in human D54 GB cell line both *in vitro* and *in vivo*, demonstrating the role of this receptor in mediating the RLR tumor cells response to IR (Ranoa et al., 2016). On the contrary, LGP2 enhanced by IR protects the human D54 GB cell line from the cytotoxic effect induced by radiotherapy. Indeed, high LGP2 expression levels are associated with poor clinical outcome in GB patients (Widau et al., 2014). Unlike RIG-I, which represents a powerful resource for the induction of both innate immune response and apoptosis after IR treatment, MDA5 does not significantly contribute to the promotion of these processes (Ranoa et al., 2016).

To date, several early clinical trials based on the use of the synthetic RLRs agonist p(I:C) stabilized with poly-l-lysine and carboxymethylcellulose [p(I:C-LC)] in combination with radiation and TMZ in adult GB patients show an impressive increase of OS (Butowski et al., 2009; Rosenfeld et al., 2010). Further, p(I:C-LC) has also been used as a tumor peptide-based vaccine adjuvant in low-grade glioma patients achieving promising results (Okada et al., 2015).

Overall, these findings suggest the potential of integrating radiotherapy and/or chemotherapy and immunotherapy based on the activation of RIG-I for the development of new clinical perspectives for GB therapy.

## Conclusion

The development of novel and more effective treatments represents a dramatic therapeutic emergency for GB patients. In the last years, many clinical trials testing alternative therapeutic approaches for GB, including immuno- and targeted molecular-therapy have been launched. Unfortunately, the clinical response has been mild to moderate at best and observed on a very limited number of patients. The presence of dysfunctional and deregulated immune cell subpopulations constitutes a major challenge in the development of alternative immunotherapies in the treatment of GB.

GB patients and preclinical models have reported several mechanisms of systemic immunosuppression: the sequestration of T cells in the bone marrow, the expansion of T-regulatory (Treg) cells which are responsible for immune tolerance and promotion of tumor growth, the suppression of natural killer (NK) cells, and the increased expression of tumor-associated macrophages (TAMs; Wiendl et al., 2002; Gabrilovich et al., 2012; Chen and Hambardzumyan, 2018). The high heterogeneity of GB appears to reflect distinct GB immune subsets based on the molecular signature (Luoto et al., 2018). A number of evidence has underlined that specific genetic alterations or epigenetic signatures can be associated with a better response to immunotherapy, and could help for selecting subgroups of GB patients (Parsa et al., 2007; Rutledge et al., 2013; Berghoff et al., 2017; Gangoso et al., 2021).

Recently, approaches aimed to activate intrinsic cellular immunity in the TME have acquired great interest, based on the capability of the RLRs signaling to induce cancer cell apoptosis while orchestrating innate and adaptive immune responses against tumor antigens. In particular, the use of synthetic RLRs mimetics is being investigated in preclinical and early clinical studies of several hematological and solid tumors (Sabbatini et al., 2012; Okada et al., 2015; Dillon et al., 2017; Mehrotra et al., 2017). However, the potential success of RLR agonists for the treatment of GB requires that some issues have to be warmly considered, such as the possible on-target induction of autoimmunity or the induction of a cytokine “storm” that could pose a threat to patient safety (Trinchieri, 2010; Buers et al., 2016; Lee-Kirsch, 2017). Another major obstacle to the widespread use of RLRs agonists in cancer treatment is their delivery to tumor cells. Palmer et al. (2018) focused on this aspect with the aim to generate stable, specific, and potent RIG-I ligands that retain functionality *in vivo*.

Although future works are needed for the translation of RLRs agonists in clinical practice, the multifaceted mechanisms by which they eliminate tumor cells represent a promising weapon to fight this devastating and incurable tumor.

## Author Contributions

FB and IB performed the literature research and drafted a first version of the manuscript. LDM and PI supervised and coordinated the work as well as wrote and edited the manuscript. All authors contributed to the article and approved the submitted version.

## Conflict of Interest

The authors declare that the research was conducted in the absence of any commercial or financial relationships that could be construed as a potential conflict of interest.
